# *Agrobacterium*-Mediated Stable Genetic Transformation of *Populus angustifolia* and *Populus balsamifera*

**DOI:** 10.3389/fpls.2016.00296

**Published:** 2016-03-09

**Authors:** Priti Maheshwari, Igor Kovalchuk

**Affiliations:** Department of Biological Sciences, University of Lethbridge, LethbridgeAB, Canada

**Keywords:** *Agrobacterium tumefaciens*, luciferase, poplar, *Populus angustifolia*, *Populus balsamifera*, regeneration, stable transformation

## Abstract

The present study demonstrates *Agrobacterium tumefaciens-*mediated stable genetic transformation of two species of poplar – *Populus angustifolia* and *Populus balsamifera*. The binary vector pCAMBIA-Npro-long-Luc containing the luciferase reporter gene was used to transform stem internode and axillary bud explants. Putative transformants were regenerated on selection-free medium using our previously established *in vitro* regeneration method. Explant type, genotype, effect of pre-culture, *Agrobacterium* concentration, a time period of infection and varying periods of co-culture with bacteria were tested for the transformation frequency. The highest frequency of transformation was obtained with stem internode explants pre-cultured for 2 days, infected with *Agrobacterium* culture at the concentration of OD_600_ = 0.5 for 10 min and co-cultivated with *Agrobacterium* for 48 h. Out of the two genotypes tested, *P. balsamifera* exhibited a higher transformation rate in comparison to *P. angustifolia*. The primary transformants that exhibited luciferase activity in a bioluminescence assay under the CCD camera when subjected to polymerase chain reaction and Southern blot analysis revealed a stable single-copy integration of *luc* in their genomes. The reported protocol is highly reproducible and can be applied to other species of poplar; it will also be useful for future genetic engineering of one of the most important families of woody plants for sustainable development.

## Introduction

Poplars are deciduous trees of the willow family (*Salicaceae*) with over 30 species distributed in the temperate areas of the Northern hemisphere that differ in leaf size and shape as well as in the color of the bark, etc. They have a short life span but are one of the fastest growing temperate trees – they can grow 5–10 feet per year (Stanturf et al., 2001). They are known as “the trees of the people” because of their use to build homes, tools, to make medicines, and protect river banks since 1000s of years (Gordon, 2001). Due to high elasticity, they are most important tree species in the snowboards and musical instrument industry. Their wood is also used for panel painting which includes the famous Mona Lisa. Today they not only provide raw wood for construction, pulp and paper industries but are used for environmental benefits, such as the reforestation of lowlands, reduction in sediment run-off, phytoremediation of contaminated soils, habitat for wildlife, etc. (Rishi et al., 2001). Besides, poplars are very desirable as of lately for biofuel production because of their fast growth, producing a significant amount of biomass in a short period of time. Their characteristics such as a small genome size, a rapid juvenile growth, a prolific sexual reproduction, and the availability of extensive genetic maps, have enabled *Populus* tree species to emerge as a model for woody plants (Sterky et al., 1998; Confalonieri et al., 2003).

Apart from the aforementioned uses, there is a tremendous potential for engineering *Populus* species with improved and novel traits, for instance, the development of varieties with tolerance toward various biotic (viz. herbicide, disease, and insect resistance) and abiotic stresses (e.g., drought, salinity, or frost tolerance helping them flourish in difficult locations such as places where there is high air pollution), the production of plants suited for wider applications in the bioproduct industry. The already available examples include a salt-tolerant variety of *P. tomentosa* that has been engineered to be grown in areas of saline land (Du et al., 2012). Their natural ability to detoxify heavy metals can be enhanced by making them store these metals in their leaves. Varieties with a reduced lignin content by creating knockouts of CAD (cinnamyl alcohol dehydrogenase) and caffeic acid *O*-methyltransferase (COMT), key enzymes in the lignin monomer synthesis pathway, will render them lucrative by reducing the cost of pulp production due to an increased digestibility (Polle et al., 2013). This will not only reduce the cost of paper manufacture but would open the door to cheaper biofuels and wood pulp that require only a fraction of the energy and chemicals, rendering the entire process environmentally friendly. The genetically engineered poplars also offer a very good means for the sustainable production of specialty chemicals, e.g., 2-phenylethanol, thus finding the use in important commodity materials, including an aviation fuel component (Costa et al., 2013). These engineered *Populus* will be a source of biofuels, biodegradable plastics and biopolymers that are more tolerant to heavy metals and would meet the growing demand for sustainable, renewable sources of biomass. A detailed account of various genetically engineered poplar species and traits already developed have been provided by Confalonieri et al. (2003).

Various *Populus* species have been transformed by *Agrobacterium tumefaciens* (Parsons et al., 1986; Fillatti et al., 1987; De Block, 1990; Leple et al., 1992; Kajita et al., 1994; Confalonieri et al., 1995, 2000, 2003; Tzfira et al., 1996; Balestrazzi et al., 2000; Han et al., 2000, 2013; Dai et al., 2003; Li et al., 2008, 2009; Yevtushenko and Misra, 2010; John et al., 2014). The transformation has been carried out using various explants, such as stem internodes, hypocotyls, cell suspension cultures, petioles, stem segments, and leaves (Confalonieri et al., 2003). For the increased genetic transformation of *Populus*, the development of robust protocols suitable for the transformation of newer species is necessary. The current study demonstrates a stable transformation of two important species, *Populus angustifolia* and *P. balsamifera*, which has not been reported before. Our previously developed method for *in vitro* regeneration of these two species was used for the regeneration of putative transformants (Maheshwari and Kovalchuk, 2011). Since the factors effecting *in vitro* regeneration have already been optimized by us in that study (including effects of genotype, explant type, hormone composition, and combinations), the current study was extended to test variables that affect transformation efficiency with the aim to develop an efficient and reproducible method that could also be applied to other species. These include genotype, bacterial concentration, explant type (stem and axillary buds), preculture in an auxin-rich medium prior to shoot regeneration, infection duration and co-cultivation time. The luciferase reporter gene was used for visual confirmation of transgene integration. Southern blot analysis showed single-copy integration of T-DNA into the genome of transgenic plants. We demonstrated that the transformation efficiency improved significantly by multiple independent factors: explant preculture in an auxin rich-medium for 2 days, *Agrobacterium* infective suspension with an OD_600_ of 0.5, an infection time of 15 min, a reduced co-cultivation period for 48 h and that stem internodal explants and *P. balsamifera* exhibited the highest transformation efficiency. We have successfully established an efficient method of transgenic plant regeneration in two genotypes of poplar that can be used for the routine transformation to produce elite varieties and will add to various transformation methods available for other genotypes.

## Materials and Methods

### Donor Plant Material

Fifteen clones of each *P. angustifolia* and *P. balsamifera* were derived from dormant buds and were grown in the greenhouse at a temperature of 25–28°C and a 16-h light/8-h dark photoperiod. They were fertilized every alternate week with Terico fertilizer (N, P, K, and S in the ratio of 20:10:10:10; Western Cooperative Fertilizer, Ltd.). These plants served as donor plantlets and were used for obtaining explants for transformation studies.

### Explant and Media Preparation

Stem internodes and axillary bud explants (size ∼ 0.5–1.0 cm) were obtained from the donor plants. These were washed thoroughly under running tap water for 30 min. They were then sterilized for 15 min in a 10% (v/v) bleach solution followed by treatment with 70% ethanol for 1.5 min. Finally, the explants were rinsed four times with sterile distilled water and subsequently cultured on Murashige and Skoog (MS) basal medium (Murashige and Skoog, 1962) supplemented with 3% (w/v) sucrose, 0.2% (w/v) myo-inositol, 0.25% (w/v) MES and gelled with 0.8% (w/v) agar. TDZ (Thidiazuron), BA (N6 Benzyl Adenine) and zeatin in combination with auxins NAA (1-naphthalene acetic acid) and 2,4-D (2,4-dichlorophenoxy acetic acid) in different concentrations were used as plant growth regulators in the basal medium, as already established by us previously (Maheshwari and Kovalchuk, 2011). The pH of the medium was adjusted to 5.70 ± 0.05 with 0.1 N NaOH or 0.1 N HCl before autoclaving at a pressure of 1.06 kg cm^-2^ for 20 min. Carbenicillin (500 mg/l) and cefotaxime (100 mg/l) were added to the medium post-autoclaving to prevent the loss of explants caused by the contamination due to the growth of endogenous microbes.

### Transformation Vector and *Agrobacterium* Culture

*Agrobacterium* strain GV3101 harboring the helper plasmid pPM6000 and pCAMBIA-Npro-long-Luc as a binary vector was used for the transformation. The binary vector was comprised of a T-DNA cassette containing the active *luciferase* (*luc*) reporter gene driven by the *N-gene* promoter and the *hph* gene conferring resistance to the antibiotic hygromycin. A single colony of this *Agrobacterium* strain was grown overnight at 28°C in 10 ml of liquid yeast extract peptone (YEP) medium in the presence of appropriate antibiotics (rifampicin, 25 mg/l; kanamycin, 50 mg/ml; gentamycin, 25 mg/ml). This overnight culture was used to inoculate 250 ml of liquid YEP medium supplemented with the abovementioned antibiotics, 200 μM acetosyringone (AS) and incubated at 28°C until O.D A_600 nm_ of 1.0–1.5 was reached. The cells were collected by centrifugation. The pellet was washed by re-suspending in MS liquid basal medium; pH 5.6 (resuspension solution) followed by centrifugation at 4000 rpm for 20 min. Suspensions of *Agrobacterium* of varying concentrations (O.D A_600 nm_ = 0.2, 0.5, 0.8, and 1.0) were prepared in resuspension solution. 200 μM of acetosyringone was added to these different cell suspensions and incubated at 28°C for another 2 h with constant shaking at 150 rpm and was thereafter used for inoculating the explants. A previous research has shown that the transformation efficiency increases in the presence of AS (induces the expression of *vir* genes) in the co-cultivation medium (Nilsson et al., 1992). Hence, in current studies, AS was incorporated in the *Agrobacterium* culture medium as well as in the suspensions used for the inoculation of explants.

### *In Vitro* Regeneration System

#### Stem Internodes

Our previously established *in vitro* regeneration system from stem explants of *P. angustifolia* and *P. balsamifera* was employed for this study (Maheshwari and Kovalchuk, 2011). The sterilized stem internode explants that were split longitudinally were exposed to a four-step culture system as described previously in our study. In the first step of callus induction, the explants were given pre-treatment on auxin-rich medium by culturing on the callus induction medium (CIM; MS basal medium supplemented with 0.2 mg/l 2,4-D) for 7 days. These were then transferred to the shoot induction medium (SIM; MS basal medium supplemented with 0.02 mg/l TDZ). Multiple shoot buds regenerated on this medium were then separated and sub-cultured in the same medium every 2 weeks for further proliferation. Calli with these regenerated shoot buds were then sub-cultured on the MS basal medium supplemented with reduced levels of TDZ (0.001 mg/l) to further increase the frequency of shoot regeneration. Once shoot buds were obtained, they were transferred to the shoot elongation medium (SEM) with the reduced cytokinin levels (0.05 mg/l BA) to favor shoot elongation and proliferation. Shoots larger than 20 mm in size were separated and transferred to the root induction medium (RIM; the half-strength MS basal medium supplemented with 2.0 mg/l IBA) in magenta boxes. The entire process of shoot regeneration was completed in a short duration of 10 weeks. The roots regenerated within 2 weeks, after that, the rooted plants were washed under tap water and subjected to hardening and/or acclimating by transfer into the tubes filled with distilled water. These tubes were placed in a growth chamber for 1 week. Thereafter, they were transplanted into the pots filled with a mixture of sterile vermiculite and sand (1:1) and maintained in the growth chamber. A high relative humidity (80–90%) was maintained by covering the pots with plastic bags. Once established in soil, the plants were transferred to the greenhouse. All cultures were maintained at 25 ± 2°C and a 16/8 h photoperiod under cool white fluorescent tube lights (40–60 mM/m^2^/s) at 60–70% relative humidity unless otherwise stated.

#### Axillary Buds

The sterilized axillary bud explants (0.1–0.5 cm long) were cultured in the MS basal medium supplemented with 0.5 mg/l BA and incubated at 25 ± 2°C and a 16/8 h photoperiod under cool white fluorescent tube lights (40–60 mM/m^2^/s) at 60–70% relative humidity unless otherwise stated. Shoot buds elongated within 10 days. These are then transferred to SEM followed by subsequent steps, as described in regeneration from stem explants.

### *Agrobacterium* Transformation and Regeneration of Putative Transformants

Various factors affecting the transformation efficiency were tested in the study: species, explant type, the effect of preculture on auxin-rich medium, bacterial concentration, infection duration, and co-cultivation time. For this purpose, sterile explants (intermodal stem explants and axillary buds) of both *P. angustifolia* and *P. balsamifera* were pre-cultured for 0, 2, 4, and 7 days in CIM. These were then infected by soaking them in *Agrobacterium* suspensions of different concentrations (OD A_600 nm_ = 0.2, 0.5, 0.8, and 1.0) for the varying periods of time (5, 10, 15, 30 min, 1, 3, 5 h) with a continuous shaking at room temperature in the dark. The longitudinal splitting of the internodal stem explants and incisions in axillary buds enhanced *Agrobacterium* infection by exposure of wounded sites (Maheshwari and Kovalchuk, 2011). The inoculated explants were then co-cultivated on CIM at 25 ± 2°C in the dark for 1–3 days. These were then washed with sterile distilled water to remove excess bacteria, blotted dry on a sterile filter paper and subsequently transferred to CIM. After incubation for another 5 days on CIM for a further growth, the explants were then transferred to SIM for the induction of shoot buds. No selection pressure was applied in the culture medium because it was observed that hygromycin was detrimental to the regeneration of callus and shoot buds. The 0.5–1.0 cm long shoots were transferred to SEM. The elongated shoots were excised and transferred to RIM. After ∼2 weeks of root induction, the plantlets were transplanted to soil. When established, plantlets were analyzed for the presence of a transgene and its expression. All the mediums used after *Agrobacterium* co-cultivation were supplemented with 500 mg/l carbenicillin and 100 mg/l cefotaxime to control the growth of endogenous bacteria and *Agrobacterium* unless otherwise stated.

### Analysis of Putative Transformants

#### Visualization of the Luciferase Reporter Gene

The expression of the *luciferase* gene in the explants post-transformation after 2–4 days as well as in the putative transformants (small plantlets, leaves, and stems of the regenerated T0 plants) were visualized with a bioluminescence assay using a CCD camera (Gloor Instruments; Basel, Switzerland). A solution containing 0.5 mM beetle luciferine (Promega) and 0.05% Tween-80 (luciferase substrate) was sprayed on the plant material to be tested which then was incubated in the dark for 30–45 min. The leaves were then photographed in the dark. The plants exhibiting luciferase expression were considered transgenic and were further analyzed by molecular methods. The number of plants expressing the *luc* gene was related to the total number of regenerated plants to obtain the transformation frequency.

#### Molecular Analysis

##### DNA extraction and PCR analysis

Genomic DNA was isolated from the leaves of wild-type and the regenerated putative transformants of *P. angustifolia* and *P. balsamifera* using the *Gene Elute Plant Genomic DNA Miniprep Kit* (Sigma) according to the manufacturer’s instructions. The DNA quality and yield were checked by running the samples on a 0.8% agarose gel and by recording the absorbance at A_260_ and A_280_. End-point polymerase chain reaction (PCR) analysis was performed on the isolated DNA to amplify a 575 bp fragment of the *luc* gene by using luciferase gene specific primers, LucF (5′-ATTTATCGGAGTTGCAGTTGCGCC-3′) and LucR (5′-CCAGCAGCGCACTTTGAATCTTGT-3′). The pCAMBIA NLUC plasmid was used as a positive control template while the wild-type genomic DNA obtained from both species served as a negative control. The PCR reaction was setup in a total volume of 25 μl containing 200 ng genomic DNA, 2.5 μl PCR buffer, 1 μl 2.5 mM dNTPs, 2.5 μl 25 mM MgCl_2_, 10 pM of each primer and 1.5 U *Taq polymerase*. The PCR program consisted of initial denaturing at 94°C for 5 min, followed by 40 cycles at 94°C for 1 min, 66°C for 30 s, 72°C for 30 min, and a final extension for 10 min at 72°C. The amplified PCR product was visualized and photographed using the gel documentation system UVP after electrophoresis on a 1% (w/v) agarose gel containing 0.5 μg/ml ethidium bromide. This amplification would confirm a stable integration of T-DNA into the cells.

##### Southern blot hybridization

Southern blot analysis was carried out to detect the number of T-DNA copies inserted in the plants that indicated the presence of a transgene. The genomic DNA from the wild-type *P. angustifolia* and *P. balsamifera* served as a negative control, while the binary plasmid was used as a positive control. 10 μg of the total genomic DNA prepared from the negative controls as well as from the transformants that indicated the presence of a transgene from both species was digested overnight with 10 U of the restriction enzyme *Nde*I (New England Biolabs, Beverly, MA, USA) in a final volume of 1000 μl. The positive control plasmid pCAMBIA NLUC (1 μg) was also digested overnight with 2 U of *Nde*I. The DNA was separated by electrophoresis on a 0.8% agarose gel in TAE buffer. The gel was then treated in 0.4 M NaOH for 30 min followed by 20 min in 0.25 M HCl. The DNA was then blotted onto a positively charged nylon membrane (Roche, Mannheim, Germany) by vacuum transfer. The DNA was crosslinked on the membrane by exposure to the UV light. The subsequent hybridization and washes of the membrane were performed according to the manufacturer’s instructions (DIG Application Manual for Filter Hybridization; Roche, Mannheim, Germany). A 575 nt probe was synthesized using the PCR DIG Probe Synthesis Kit (Roche, Mannheim, Germany) according to the manufacturer’s instructions using the pCAMBIA NLUC plasmid as a template and the above-mentioned luciferase-specific primers (LucF and LucR). The membrane was hybridized with this DIG-labeled probe overnight at 42°C in hybridization buffer. High stringency membrane washes were carried out at 65°C. Chemiluminescence detection was performed by the Dig luminescent detection kit for nucleic acids (Roche) with Amersham Hyperfilm ECL (GE Healthcare).

### Statistical Analysis

Fifteen explants were transferred to a 90 mm diameter Petri plate. All experiments were carried out in triplicates with ten plates per treatment, 15 explants per each plate. The number of explants that formed shoot buds, the number of shoot buds per explant, and the number of regenerated putative transformants that exhibited the presence of a transgene were recorded to calculate the frequency of shoot regeneration and transformation. The statistical analysis was performed using MS Excel software and JMP 5.0 software (SAS Institute, Inc.) and R (version 2.10.1) statistical package (freeware). In all cases, the mean and standard error or the standard deviation was calculated, and the statistical significance of the experiment was confirmed either by the two-tailed paired Student’s *t*-test with *a* = 0.01 (comparing data from two treatments) as well as by a single-factor ANOVA (comparing data from three or more treatments).

## Results and Discussion

### Transient Expression Analysis

**FIGURE 1:**
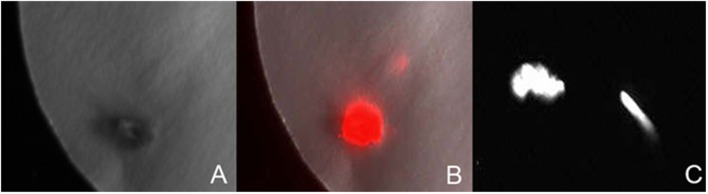
**Transient luciferase expression.** **(A)** The image of *Populus angustifolia* axillary bud explant taken in light. **(B)** The dark image of an explant shown in image **(A)** exhibiting chemiluminescence. **(C)** Chemiluminiscent image of axillary bud and stem explant of *P. balsamifera*.

Luciferase reporter gene expression was employed to monitor the transient expression for studying the effect of various factors for the optimization of transformation of *P. angustifolia* and *P. balsamifera*. Luciferase expression in the explants was analyzed after spraying the luciferase substrate (**Figure 1**). Both stem internodes and axillary bud explants were used to study the effect of single factors (pre-culturing of extracts, *Agrobacterium* concentration, etc.) or their combination on the regeneration of stable transformants.

#### The Effect of Preculture of Explants

Auxin pre-treatment of explants for 7 days before transferring to SIM for shoot regeneration was shown to enhance shoot regeneration competence in *P. angustifolia* and *P. balsalmifera* explants (Maheshwari and Kovalchuk, 2011). We tested this effect of preculture in CIM on *Agrobacterium* transformation. No significant difference in the transient expression of the luciferase reporter was recorded between stem internodes and axillary bud explants pre-cultured for a period of 0–7 days in both *P. angustifolia* and *P. balsamifera.* However, it was noted that 2 weeks after treatment with *Agrobacterium*, the explants that were pre-cultured for 1–7 days survived the infection better in comparison to those that were not pre-cultured. It was previously suggested that preculture accelerates the rate of T-DNA incorporation in cells by increasing their competence rather than increasing the rate of DNA transfer from *Agrobacterium*, allowing a greater survival of transformed cells (Binns and Thomashow, 1988). Similar findings were observed in a variety of cottonwood species (De Block, 1990; Balestrazzi et al., 2000; Han et al., 2000).

Stem internodal explants of *P. angustifolia* and *P. balsamifera* pre-cultured for 1 day survived the infection better by ∼45.23 and ∼52.63%, respectively, in comparison to those that were not pre-cultured. The survival frequency increased with increasing the duration of preculture, and it was the highest in the explants pre-cultured for 4 days; the pretreated *P. angustifolia* and *P. balsamifera* explants survived infection better than non-pretreated by ∼68.12 and ∼73.14%, respectively. An increase in the time of preculture beyond 4 days resulted in a decrease in the frequency of the transient expression, which was most likely due to wound healing in the explants as it was also shown in a previous study on *P. tremula* (Tzfira et al., 1997). Also, a preculture period of up to 4 days significantly increased the rate of callus formation in the transformed stem internode explants as well as shoot elongation in the axillary bud explants in comparison to those which were directly infected. This is in agreement with the results of our previous study where we demonstrated that auxin pre-treatment enhances cell division (Maheshwari and Kovalchuk, 2011). A preculture period of 2 days appeared to be optimal, demonstrating a fivefold higher callus regeneration efficiency as compared to a period of 0 day, or 3.8-fold higher as compared 4 days and 1.2-fold as compared to 7 days. We observed that the explants cultured for up to 4 days better coped with stress of *Agrobacterium* infection in comparison to the explants which were directly infected without a preculture phase; the plants infected directly exhibited a greater degree of necrosis (data not shown).

#### The Effect of *Agrobacterium* Concentration

*Agrobacterium* suspensions of different concentrations (OD A_600_ = 0.2, 0.5, 0.8, and 1.0) were used to infect explants for 30 min, and the effect on the transformation frequency was analyzed. The lowest transient transformation frequency of 63–72% was observed in plants incubated in Agrobacteria with OD_600_ = 0.2, whereas the highest one of 92–95% was observed when OD_600_ = 0.5 was used. At increased concentrations (OD_600_ ≥ 0.8), the transformation frequency was also high (82–98%), but due to an increased contamination of the explants with *Agrobacterium* overgrowth, most explants died due to necrosis. The bacterial concentration has been shown to affect transformation frequencies in a few other studies as well. Kim et al. (1997) suggested the bacterial concentration of 5–6 × 10^8^ cells/ml as an optimal one for the transformation. Similarly, 1.2 × 10^9^ cells/ml generated the efficient transformation of *P*. *alba* and *P*. *nigra* (Confalonieri et al., 1994).

#### The Role of Duration of Infection

**FIGURE 2:**
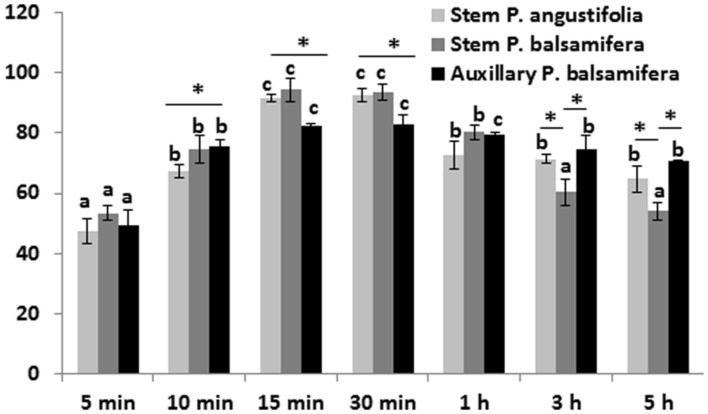
**The transient transformation frequency of various poplar explant types after different durations of infection with *Agrobacterium*.** Stem internodal and axillary bud explants were infected by soaking them in *Agrobacterium* suspensions of concentrations at OD A_600 nm_ = 0.5 for varying periods of time – 5, 10, 15, 30 min, 1, 3, 5 h with a continuous shaking at room temperature in the dark. Transient expression of the luciferase gene was analyzed after 48 h of incubation. The data are the average of three experiments with SD, each consisting of 100 explants. Letters indicate significance difference (*p* < 0.01) between soaking times, calculated separately for each genotype/explant combination (similar letters over the bars mean no significant difference). ^∗^ Indicate a significant difference in the regeneration efficiency calculated between different species/explant types (*p* < 0.05).

At OD_600_ ranging from 0.4 to 0.5, the suitable incubation time of explants in the bacterial suspension ranged from 15 to 30 min (the transient transformation efficiency of 82–96%; **Figure 2**). Previous studies by Confalonieri et al. (1994) showed that the optimal procedure involved dipping of leaf disks into a bacterial suspension (7 × 10^8^ cells/mL) for 20 min. Similarly, Han et al. (2013) demonstrated that at OD_600_ ranging from 0.8 to 1.0, the suitable incubation time of explants in the bacterial suspension ranged from 20 to 30 min, whereas according to Movahedi et al. (2014), the highest transformation efficiencies in the *Populus* hybrid clone “Nanlin895” (*P. deltoides* × *P. euramericana*) were obtained from the immersion for 120 and 150 min and were not significantly different from each other. This indicates that different poplar species benefit from different durations of infection with Agrobacteria.

#### Co-cultivation Length

In addition to preculture, *Agrobacterium* concentration and infection time, the transient transformation frequency was also influenced by the co-cultivation time after the infection. We selected three different durations for co-cultivation – 1–3 days. Co-cultivation for 72 h resulted in 100% efficiency of transient transformation, but it also resulted in high bacterial overgrowth and necrosis of explants. A similar observation of an increased bacterial growth leading to a reduced regeneration of plantlets has been made in Aspens (Tzfira et al., 1997). Co-cultivation for 24 h resulted in a transformation frequency ranging from 58 to 71%, whereas that for 48 h resulted in 81–89% transformation frequency (**Table 1**). Importantly, the level of necroses was substantially reduced in all explant types co-cultivated for up to 48 h. Therefore, it was concluded that co-cultivation for 48 h is the most optimal. This was in agreement with the work on other genus of poplar where it was shown that co-cultivation for at least 48 h was considered essential (Howe et al., 1994; Tsai et al., 1994; Han et al., 2000; Du et al., 2012).

**Table 1:** The transient transformation frequency of various types of poplar explant after different durations of co-cultivation with *Agrobacterium*.

Duration of co-cultivation	*P. angustifolia*		*P. balsamifera*	
		
	Stem internode explants	Axillary bud explants	Stem internode explants	Axillary bud explants
24 h	58.33 ± 3.51^a^	70 ± 3^b,c^	66 ± 2^b^	71.33 ± .08^c^
48 h	86.33 ± 3.78^d,e^	84 ± 1.73^d^	89.66 ± 2.51^e^	81 ± 3.60^d^
72 h	100^f^	100^f^	100^f^	100^f^


*The table shows the average percentage of LUC-positive sterm internodes or axillary bud explants of *Populus angustiofolia* or *P. balsamifera* regenerated after co-cultivation with *Agrobacterium* suspension for varying periods of time – 4, 48, and 72 h. The data are the average of three experiments with SD, each consisting of 100 explants. Different letters indicate that means are significantly different at the 0.05 probability level. There is also a significant difference between the transient transformation frequency for different duration of co-cultivation within the same explant group at *p* < 0.05.*

### Tissue Culture and the Regeneration of Transformants

**FIGURE 3:**
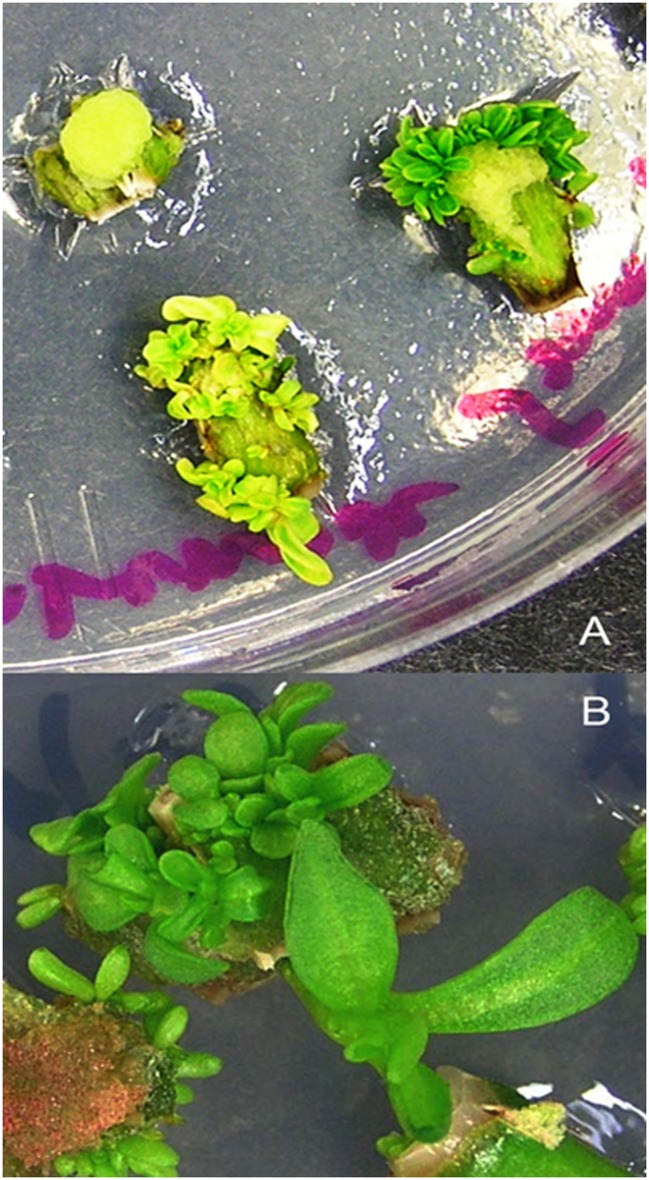
**Regeneration of putative transformants from stem explants of *P. angustifolia*.** **(A)** Shoot proliferation in SIM (MS basal medium supplemented with 0.02 mg/l TDZ). **(B)** Shoot elongation in SEM (MS medium containing 0.05 mg/l BA) after 4 weeks of culture. Regeneration was carried out in a selection free medium.

**FIGURE 4:**
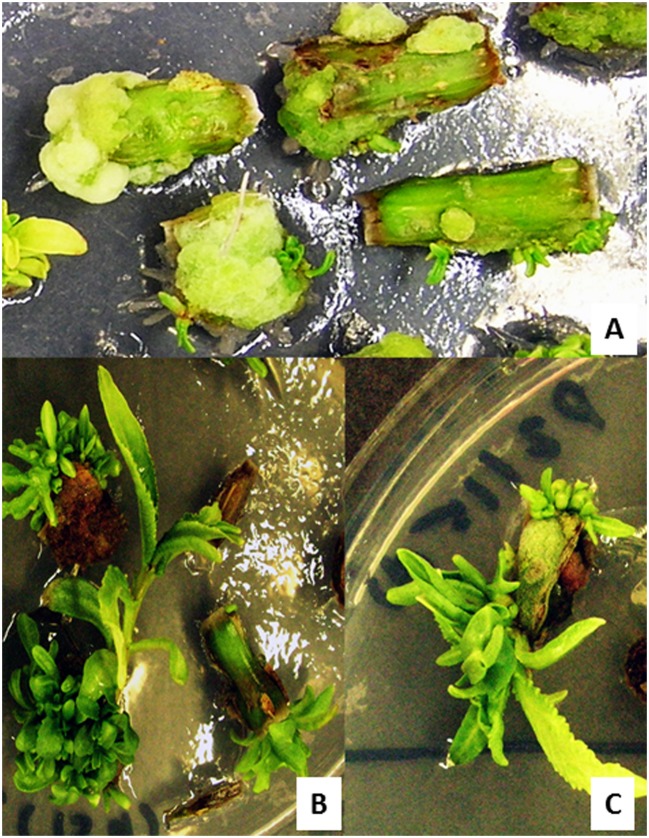
**Regeneration of putative transformants of *P. balsamifera* from stem internodal explants in MS medium containing 0.02 mg/l TDZ.** **(A)** Shoot bud induction from a callus after 2 weeks of culture. **(B,C)** Formation of multiple shoots and shoot elongation after 4 weeks of culture. Regeneration was carried out in a selection free medium.

**FIGURE 5:**
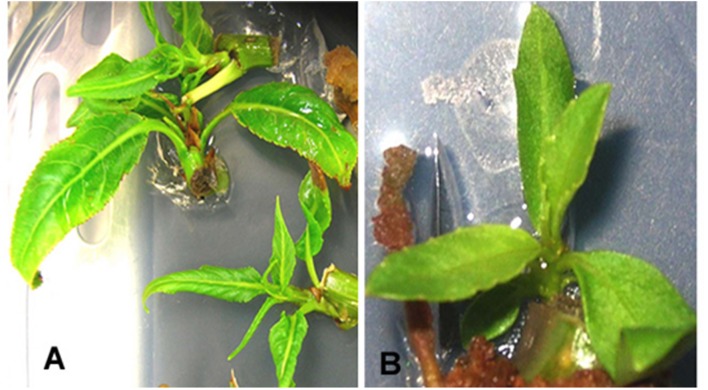
**Proliferation of putative transformants from axillary bud explants after 4 weeks of culture in MS basal medium supplemented with 0.5 mg/l BA.** **(A)** *P. balsamifera*. **(B)** *P. angustifolia.* Regeneration was carried out in a selection free medium.

Transient transformation experiments allowed establishing optimal conditions for a stable transformation. Stem internode explants that were split longitudinally and axillary buds pre-cultured for 2 days on CIM and shoot regeneration medium, respectively, were infected with *Agrobacterium* culture harboring the luciferase reporter gene containing a binary vector at an OD_600_ = 0.5 for 15 min. These were then co-cultivated for a period of 48 h on CIM (for stem explants) and shoot regeneration medium (for axillary bud explants) respectively. Next, the explants were thoroughly washed in sterile distilled water containing 100 mg/l carbenicillin and dried on a sterile filter paper. Since the stem indernode explants were already incubated on CIM for 4 days (2 days during preculture and then 2 days during co-cultivation), they were transferred to CIM supplemented with carbenicillin (500 mg/l) and cefotaxime (100 mg/l) and cultured for another 2 days in accordance to our previous findings where exposure to auxin for 6 days was found to exhibit a maximum frequency of shoot induction prior to cytokinin treatment (Maheshwari and Kovalchuk, 2011). These were transferred to SIM supplemented with carbenicillin (500 mg/l) and cefotaxime (100 mg/l) for the regeneration of multiple shoot buds which were then separated and sub-cultured every 2 weeks finally to be transferred to SEM for obtaining elongated shoots suitable for root regeneration (**Figures 3** and **4**). Similarly, the axillary bud explants were transferred to the antibiotics containing (carbenicillin 500 mg/l and cefotaxime 100 mg/l) shoot regeneration medium where shoots proliferated in 4 weeks (**Figure 5**).

Carbenicillin and cefotaxime incorporated in the culture medium were quite effective in controlling the *Agrobacterium* overgrowth without affecting the rate of shoot induction. Instances of sporadic outgrowth of the bacterium were successfully controlled by washing the regenerating tissue with sterile distilled water containing 100 mg/l carbenicillin and transferring it to a fresh culture medium containing the antibiotics. The incorporation of hygromycin in the culture medium resulted in browning followed by the death of explants, hence a selection free regeneration was carried out.

The regeneration efficiency from stem internodal explants was 44.4 ± 1.83 and 68.89 ± 1.28% for *P. angustifolia* and *P. balsamifera*, respectively (**Table 2**). The number of shoots regenerated per explant was 8.34 ± 1.12 and 11.46 ± 2.34 for *P. angustifolia* and *P. balsamifera*, respectively. Similarly, the shoot proliferation rate from axillary bud explants was 79.11 ± 0.38 and 82.63 ± 0.65% for *P. angustifolia* and *P. balsamifera*, respectively (**Table 2**). 95% of plants were successfully rooted and transferred eventually to the greenhouse for further growth.

**Table 2:** Transformation efficiency from internodal and axillary bud explants of *P. angustifolia* and *P. balsamifera* pre-cultured on CIM for 48 h and co-cultivated with Agrobacteria for 48 h.

	Regeneration efficiency, %	Shoots per explant	Luciferase expressing shoots per explant	% Frequency of transformation per explant
***P. angustifolia***				
Stem internodes	44.4 ± 1.83^a^	8.34 ± 1.12^a^	1.65 ± 0.02^a^	19.78
Axillary buds	79.11 ± 0.38^b^	3.23 ± 1.24^b^	0.1 ± 0.05^b^	3.10
***P. balsamifera***				
Stem internodes	68.89 ± 1.28^c^	11.46 ± 2.34^c^	2.9 ± 0.02^c^	25.30
Axillary buds	82.63 ± 0.65^d^	4.56 ± 2.1^d^	0.12 ± 0.04^b^	2.63


*All experiments were carried out in triplicates with ten plates per treatment, each plate carrying 15 explants. The regeneration efficiency is the percentage of explants exhibiting shoot bud formation. Different letters indicate that means are significantly different at the 0.05 probability level.*

**FIGURE 6:**
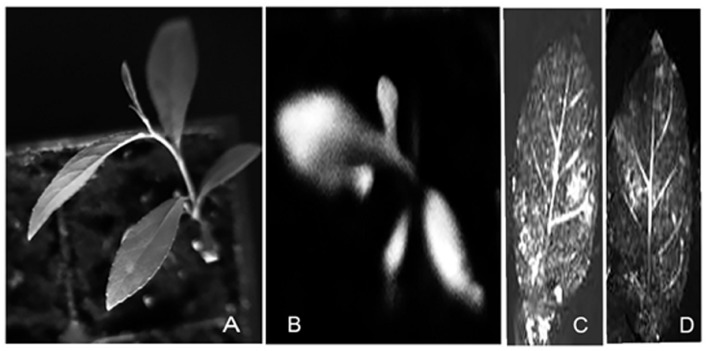
**Visualization of luciferase expression in T0 plants.** Putative transformants (T0) were visualized for luciferase expression with a bioluminescence assay using a CCD camera. Luciferase substrate was sprayed on the plant material which then photographed in the dark. **(A)** A light image of a *P. balsamifera* plantlet. **(B)** A dark image of a luciferase-positive *P. balsamifera* plantlet. **(C,D)** A dark image of leaves of luciferase-positive *P. balsamifera and P. angustifolia*, respectively.

All plants regenerated from stem and axillary buds of both *P. angustifolia* and *P. balsamifera* were tested for transgene expression (**Figure 6**). A total of 19.78 and 25.30% of plants exhibited luciferase expression amongst those regenerated from stem explants of *P. angustifolia* and *P. balsamifera*, respectively. The frequency of transformation from axillary buds was 5–6 times lower than that from internodal stem explants, 3.10 and 2.63% in *P. angustifolia* and *P. balsamifera*, respectively. The increased efficiency of regeneration from stem internodal explants over leaf and stem petioles has been reported before by De Block (1990) and Balestrazzi et al. (2000), respectively.

A reduced transformation efficiency from axillary buds indicates the difficulty of transforming meristem tissues and also that shoots obtained through direct organogenesis in poplars are mostly non-transgenic. This is in agreement to findings by Han et al. (2000) who showed that prolonged period of callus formation increases the competence for regeneration, and transgenic cells proliferate to eventually form shoots. Even though the reduced frequency of transformation was obtained from axillary bud explants, luciferase expression was observed in all organs of all transgenic lines obtained, as confirmed after spraying the entire young plantlets with the luciferase substrate followed by visualization by a CCD camera. 3 and 5% plants out of the total number of putative transformants regenerated from stem explants of *P. angustifolia* and *P. balsamifera* appeared to be chimeric and were not included in further molecular studies. In these plants, only a few leaves or parts of plantlets exhibited luciferase expression as compared to others where the entire plantlet exhibited chemiluminescence. The transgenic plants did not differ morphologically from the control non-transgenic plants and showed no signs of abnormality (data not shown).

### Molecular Analysis of Putative Transformants

**FIGURE 7:**
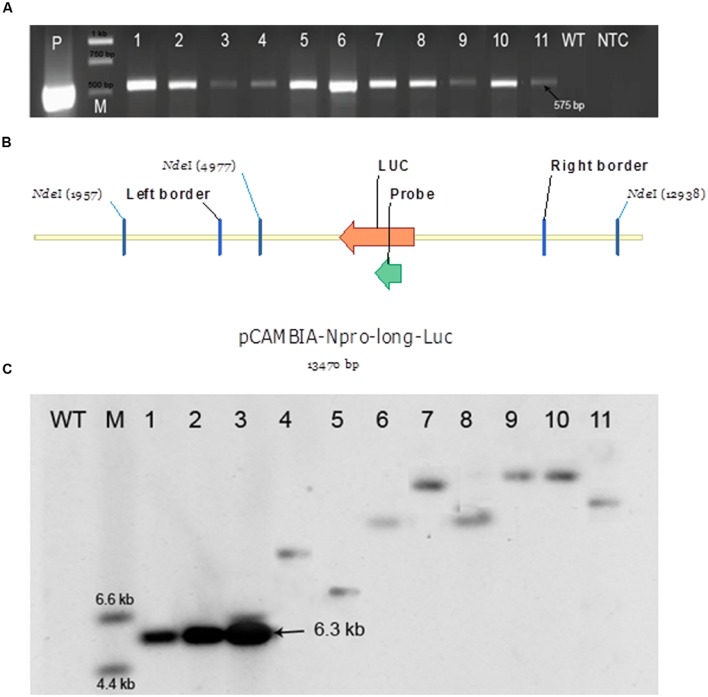
**Polymerase chain reaction (PCR) and Southern blot analyses for checking the presence of a transgene.** **(A)** PCR analysis. LucF and LucR primers were used to amplify a 575 bp fragment from the *luc* region indicating the presence of a transgene. P – a positive control (pCAMBIA:: NLUC plasmid); M – Generuler 1 kb ladder (Thermo Fisher Scientific); 1–6: six independent putative transformants of *P. angustifolia;* 7–11: five independent putative transformants of *P. balsamifera;* WT- a negative control (a wild-type plant); NTC – no template control; 575 bp – amplicon size. **(B)** Southern blot analysis of putative transformants by using a DIG labeled probe complimentary to the luciferase transgene. Design of the 13470 bp long pCAMBIA-Npro-long-Luc vector containing the T-DNA (Boyko et al., 2011). Genomic DNA of putative transformants expressing luciferase and positive for the presence of a 575 bp fragment from the *luc* region in the PCR assay was digested with the restriction enzyme *Nde*I which cuts the T-DNA at a single site. The number of fragments for each sample on the blot corresponds to the number of loci carrying the transgene. **(C)** Picture of Southern blot analysis. WT- A wild-type negative control plant; M – DIG labeled DNA molecular weight marker (Sigma-Aldrich); 1–3: 10, 100, and 250 pg of plasmid, respectively, used as a positive control; 4,5: two independent putative transformants obtained from intermodal explants of *P. angustifolia;* 6,7: two independent putative transformants obtained from axillary bud explants of *P. angustifolia;* 8,9: two independent putative transformants obtained from axillary bud explants of *P. balsamifera*; 10,11: two independent putative transformants obtained from internodal explants of *P. balsamifera*.

The regenerated plants exhibiting the luciferase activity in the bioluminescence assay during the preliminary round of screening for reporter gene expression were considered to be putative transformants and were subjected to molecular analysis. A total of 100 randomly chosen putative *P. angustifolia* and *P. balsamifera* transformants were chosen for the PCR based assay to detect the presence of a transgene as described in a previous section. The presence of the expected 575 bp fragment after PCR amplification indicated the presence of the transgene in the regenerated plants (**Figure 7A**). The plasmid was used as a positive control. No amplification of the expected size was observed in the non-transgenic control plants. All the plants that exhibited luciferase expression in the bioluminescence assay demonstrated the presence of the transgene in PCR.

Two randomly chosen PCR-positive plants derived from each stem intermodal and axillary bud explant of both *P. angustifolia* and *P. balsamifera* were further analyzed by Southern blot analysis to evaluate the complexity of integration events in the transgenic lines. Since we used a restriction enzyme (*Nde*I) that cuts T-DNA only once and does not cut through the region of probe hybridization, the number of fragments observed on Southern blot would correspond to the number of loci carrying the transgene(s). The intact T-DNA should give a fragment of at least 6.3 kb (from the *Nde*I site to the right border) which was confirmed in the blot in samples from the transgenic line and was not observed in the non-transgenic control plant. All the eight transgenic lines subjected to Southern blot analysis revealed the presence of a single copy transgenic event in both *P. angustifolia* and *P. balsamifera* (**Figures 7B,C**).

## Conclusion

Various factors were tested in order to optimize the transformation process in two different species of poplar – *P. angustifolia* and *P. balsamifera:* explant type, preculture of explant, *Agrobacterium* concentration, duration of infection, and co-cultivation time. Most of these factors proved to be crucial for obtaining the optimal transformation. We found that a prolonged preculture time (greater than 4 days) increased the concentration of bacteria (greater than OD_600_ = 0.8); the infection time of more than 30 min and a co-cultivation period of more than 48 h led to the reduction in transformation efficiency. Amongst the two different explant types tested, stem internode explants demonstrated to be the better ones for the transformation in comparison to axillary bud explants in both species, indicating that the transformation of meristematic tissues is difficult. Although, 3 and 5% of transformants regenerated from stem explants of *P. angustifolia* and *P. balsamifera*, respectively, were chimeric and those from axillary explants were not, the transformation efficiency from axillary bud explants is much lower, which makes stem explants the material of choice. The transgenic plants, as confirmed by Southern blot analysis, revealed the presence of the transgene in a single copy. The transformants showed no phenotypic difference in comparison to the control non-transgenic plant. The reported procedure is very effective and demonstrated the applicability in two different species of *Populus*. A similar approach can be applied to develop efficient methods for other poplar species and can be used successfully for genetic engineering of economically important tree species.

## Author Contributions

PM designed the experiments, performed research, analyzed data, and wrote the manuscript; IK designed the experiments, analyzed data, and wrote the manuscript.

## Conflict of Interest Statement

The authors declare that the research was conducted in the absence of any commercial or financial relationships that could be construed as a potential conflict of interest.
